# Theatrical Performance as Leisure Experience: Its Role in the Development of the Self

**DOI:** 10.3389/fpsyg.2020.01439

**Published:** 2020-06-30

**Authors:** José Vicente Pestana, Rafael Valenzuela, Nuria Codina

**Affiliations:** Department of Social Psychology and Quantitative Psychology, University of Barcelona, Barcelona, Spain

**Keywords:** leisure, leisure experience, theater, self, psychosocial intervention

## Abstract

Theater has been used in psychological intervention and as a metaphor for social life, tendencies that affect the self, highlighting how influential theatrical performance can be for individuals. Their limitations – in terms of the empowerment of the self and its authenticity, respectively – can be overcome by treating theatrical performance as a leisure experience, which considers that freedom and satisfaction play a central role in a more comprehensive understanding and development of the self. With this in mind, we present the conceptual and empirical bases of the leisure experience as an alternative conception of theatrical performance. To do so, we organized a 20 h theater exercise workshop with 16 university students (15 women, one man), aged between 18 and 21 years old (*M* = 19.06 years; *SD* = 1.06). The instruments used were: the Time Budget Technique (questionnaire about the activities carried out in the workshop, valued in relation to two items: perceptions of freedom and satisfaction); the Twenty-Statement Test (where people list characteristics of themselves – self-descriptions – related in this case to the theatrical exercises); and, as a third instrument, a combination of the other two – specifying which exercises were more closely related to the self-descriptions. The results showed that group discussion was the activity with the highest perception of freedom, followed by obstacle exercises; as regards the perception of satisfaction, the highest value was observed in the relaxations. In the case of the self-descriptions, the acquisition of practical and intellectual skills was significant, as well as emotional outlook and the expression of self-esteem. In sum, this empirical support – using instruments that invite an exploration of the self – revealed, on the one hand, which specific characteristics of the self are manifested by doing theatrical exercises and, on the other hand, which exercises – when experienced as leisure – have a more decisive impact on the self. Thus, this paper shows what aspects must be taken into account when deciding which activities to include in a psychosocial intervention addressed to the development of the self from the standpoint of theatrical performance as a leisure activity.

## Introduction

Theatrical performance includes those behaviors that, both on- and offstage, help us to understand the details of the processes of the self – which requires adequate instrumentalization to contribute to this knowledge of human behavior (Wilshire, 1991; Marcus and Marcus, 2011; Zamir, 2014). This possibility of understanding the processes of the self resides in the fact that, unlike the enlarged picture on the big screen or the reduced picture on a television or computer, “theatre is exactly the same size as life, neither larger nor smaller. Its subjects and its concerns may take on larger dimensions, but the form itself is life-size and that is how we receive it” (Finnbogadóttir, 1999).

Given this life-sized characteristic of theater, the origins of this activity can be imagined as an occasion where an agreement was made between human beings – at least two – to draw an imaginary line. At this invisible frontier, one party began to show the other something that had happened – or that could happen – at any time and place. In this scenario of interaction, the presentation of a past event (i.e., its re-presentation) or the act of anticipating something in the future (i.e., prospectively) sheds light, in the case of both parties involved, on the human capacity to transcend time and space (Petrella, 2011), this thanks to the faculty of the imagination (Rozik, 2002) and by virtue of the self – i.e., the ability to perceive oneself, even beyond the here and now (Mead, 1972).

This description condenses several issues that in one way or another – and in line with previous contributions – direct attention toward the self as a psychosocial process, which has in theater both a metaphor and a context for its analysis. This emphasis on the self does not obviate the fact that theatrical performance affects other psychological and/or social processes – such as attitudes (Hansen, 2015), learning (Webster, 2019), and emotions (Lazaroo and Ishak, 2019), to give some examples. Nonetheless, it is the self that contains the biological, intrapsychic (conscious, unconscious) and relational aspects, and “virtually any activity can be incorporated within the domain of self-psychology simply by prefixing it with ‘self-’” (Swann and Bosson, 2010, p. 591). These characteristics of the self provide an understanding of the centrality of self-referential processes in the exploration of the different kinds of theatrical performance (see Pendzik et al., 2016 for a review of these processes).

In terms of the psychological and social aspects of behavior, theatrical performance and the self have been considered primarily from the following perspectives: (1) Jungian analytical psychology; (2) behavioral-cognitive social psychology; (3) humanistic psychology; (4) symbolic interactionism in its dramaturgical aspect; and (5) critical orientations inspired by Marxism. Each of these perspectives – which we detail in the paragraphs below – has provided particular insights into the use of theater to understand self-processes. Therefore, by specifying the contribution of each of these five perspectives, we highlight the proposed contribution of this research to the existing knowledge of the subject.

**FIGURE 1:**
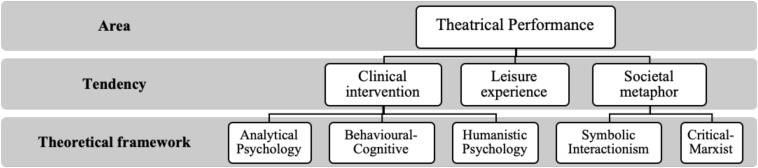
Main theoretical frameworks of theater as theatrical performance. Source: prepared by the authors following Munné (1996) and Codina (1997).

As shown in Figure 1, the first three approaches have focused on clinical interventions based around theater, while the other two offer important analyses of social life as a mise-en-scène (Figure 1). For our part, we include the perspective of theatrical performance as leisure experience (Pestana and Codina, 2017), which offers a theoretical and methodological alternative to clinical interventions and the analogies between life in society and theater. The situation of the leisure experience at a point between clinical intervention and societal metaphor is not a trivial matter, since the leisure experience embraces – at least potentially – both psychological and social factors. Furthermore, given that the leisure experience is based on perceived freedom and satisfaction, research from this perspective can shed light on the self processes involved in theatrical performance.

### Theater as a Technique in Psychological Intervention

The relevance of theater in clinical intervention is summed up by Walsh (2013, p. 73), who affirms that “theatre dialogues with therapy, positing itself as a related, if not an alternative practice for gaining insight into ourselves and our relationships.” In fact, origins of theater as an artistic genre include a psychological component (Pandolfi, 1964): the extraversion manifested by the performers in front of someone observing an action understood by convention to be fictitious. This extraversion allowed something of the inner self to emerge and, in this process, it transformed itself into an object that became part of the relationship with the other (in line with Jung, 1976a). For example, an individual who was afraid of hunting animals might have presented other individuals with a scene depicting pursuit – imaginary, fictitious – by the intended quarry; in this way, the spectators were witnesses to a staging of the said fear, which would have helped them to take this emotion into account in their relationship with the individual who had expressed something of himself. In other words, in a situation like the one in the example, as explained by Cornejo and Brik Levy (2003, p. 51), what happens is that “the individual transfers his or her internal images to the external world and the changes that are engendered in them” (authors’ translation).

This psychological component was also present in the liberation or purification proposed by Aristotle (1996, *c*. 335-323 BC/1996), under the name of catharsis, to define the public’s reaction to the tragedy. This liberation or purification has been equated with an emotional manifestation of great intensity (Jackson, 1994; Turri, 2017), and even with the expression of the psychological conflict itself (Vives, 2011). This results in a learning experience (Ávila, 2015, p. 3) because: in the tragedy it is always a question of “non-trivial” action, of significant and important action for human beings. The teaching contained in a tragedy is linked to life, to the life of human beings and what is truly meaningful to them (authors’ translation).

The importance of the role of the psychological dimension in the origins of theatrical performance and in its initial impact on the public provides an understanding of how initiatives emerged that focused on the use of theatrical activities beyond the stage – in particular, in psychological analysis and intervention. However, these initiatives do not correspond to a sole conception of the psychological. In fact, the main approaches that consider theater as a technique in psychological intervention are found to respond to the principles of Jungian analytical psychology, behavioral-cognitive psychology and humanistic psychology. As described below, each one of these theoretical approaches is important when referring to a specific model of human being – or paradigm, in the words of Munné (1996) and Codina (1997), which in the case of theatrical performance implies highlighting the complexity of human nature that may be presented onstage.

The modality of psychoanalysis derived from the contributions of C. G. Jung – i.e., Jungian *analytical psychology* describes the theater as “an institution for working out private complexes in public” (Jung, 1976b, p. 48). But then again, what are complexes and what does their observation in public entail? For Jung (Jung, 1976b) complexes are an amalgam of psychic contents that make up the self and of which we must become aware – the theater being precisely a place where this awareness can develop.

Using as an example the best known of the complexes taken from a play – the Oedipus complex (Freud, 1948) in *Oedipus Rex*, this tragedy about the King of Thebes synthesizes the difficulties involved in the relationship between children and their parents: “It is only in dreams that men find themselves in their mothers’ beds” (Sophocles, 2007, p. 107). Similarly, Carlisky (1965) applied psychoanalytic concepts to interpret other theatrical characters besides Oedipus – Hamlet, Macbeth, Sigismund – and various cinematographic works. For his part, Weissman (1967) defended the usefulness of theatrical characters as a resource that compensates for the defects that, during childhood, may have influenced the posterior self-identity and body image of young people. Thus, a character on a stage can articulate the complexes of an individual, to the point that he/she can become aware of his/her own self conflicts.

In addition to challenging the individual, Jungian analytical psychology has also considered the collective importance of theater, i.e., its relevance as the fundamental container of all humanity, as an archetype of the collective unconscious. In particular, the connection with the archetypal image of Dionysus – from whose theater festivals derived (Grimal, 2018) – leads to “a vital experience, through which a psychic rebirth takes place” (López-Pedraza, 2004, p. 35 – authors’ translation). The same author goes on to state that “there is a Dionysus in our body, who is waiting to be contacted and give us access to the richness of our emotions and feelings” (López-Pedraza, 2004, p. 45). In other words, when an individual experiences the Dionysian or theatrical archetype, this makes way for experiences that, while unrelated to everyday existence, have a revealing or even transformative impact on it:

The moment when this mythological situation reappears is always characterized by a peculiar emotional intensity; it is as though chords in us were struck that had never resounded before, or as though forces whose existence we never suspected were unloosed (Jung, 1971, p. 128).

That is to say, the mere exploration onstage of the different impulses that inhabit the body can lead to a Dionysian enjoyment that expands the self-consciousness of the individual.

As regards *behavioral-cognitive psychology*, its body of knowledge includes the cognitive abilities most apparent in people who take part in theatrical activities. For example, greater ability has been observed in faculties such as the creation of meaning (Klein, 2019), creativity (Eberle, 1974; Berretta and Privette, 1990), and memory and learning (Noice and Noice, 2002, 2006, 2013). In a more general theoretical sense, McConachie (2008, 2013) developed a proposal that defends, in the relationship between spectators and performers, the importance of mirror neurons, consisting of networks of brain cells that synchronize the transmission of both positive emotions (care, play) and negative ones (rage, panic, fear). This proposal provides a neurobiological basis for the relationship between performers and public that develops during the theatrical performance: “Theatre’s peculiar strength lies in providing *another reality* that makes it possible to work on the ability of creating relationships” (Sofia, 2013, p. 179 – in italics in the original text).

*Humanistic psychology* – the third approach in the face of the determinisms of Freudian psychoanalysis and the behavioral-cognitive focus (Moss, 2001) – boasts a solid tradition in the application of theatrical performance in psychological intervention, thanks to the work of the psychiatrist J. L. Moreno. After some initial studies, he began to speak of “Theatre of Spontaneity” (Moreno, 1947). Moreno ended up conceiving the actor’s role as “the functioning form the individual assumes in the specific moment he reacts to a specific situation in which other persons or objects are involved” (Moreno, 1994, p. IV). With this definition in mind, the implementation of psychodramatic roles, which are spontaneous reactions to imagined situations, is what enables the self to achieve a creative resolution of personal conflicts (Karp, 1994; Orkibi and Feniger-Schaal, 2019). Under this conception, individuals can experience possibilities of the self in situations not as yet experienced (Cruz et al., 2018). More recently, the combination of psychodrama and Jungian analytical psychology has allowed the observation of the expression of primary structures of human behavior and experience in general – i.e., archetypal patterns (Barz, 2014; Beach, 2014).

Mention should also be made of other approaches such as Dramatherapy and the Theater of voices (this one advocated as a tool for empirical research into the Dialogical Self). These are allied to psychodrama but not directly related to it.

Dramatherapy considers theater as an activity that makes it possible to establish links between the unconscious and the emotional processes of individuals (Jones, 1996, 2016; Emunah, 1999, 2015; Pitruzella, 2004). This piecing together is achieved, basically, through the imagination and certain alterations of the perceptual focus, i.e., through new uses of certain objects or the exploration of the self through the body (Pitruzzella, 2017). In this way, the ability to take on another self identity is stimulated. Recent reviews of the effects of dramatherapy on its participants – adults with mental health problems – underscore improved self-consciousness, empowerment and social interaction (Jaaniste, 2016; Bourne et al., 2018). In respect of the use of theater in the analysis of the Dialogical Self (see Hermans, 2006, for the details), this approach highlights the importance of the onstage exploration of I-positions, these being characters or personifications of a sort that each individual has developed within him/herself. Establishing a dialogue with the different I-positions favors the constructive integration of the various – and sometimes contradictory – realities, “with the permission and encouragement to be real” (Rowan, 2010, p. 105).

As a psychological intervention technique, theatrical performance has led to the inclusion in clinical practice of exercises whose results show transformations in the self – interpretable from different perspectives linked by a common purpose: the improved health or full recovery of those who do the theatrical exercises (Pendzik et al., 2016). Implicit in this use of theater as a psychological intervention technique is the idea that, as a general rule, the individuals who participate in this type of intervention have problems that may limit the maximum empowerment or expression of the self (Pestana, 2007): a wounded self must heal first before broadening and expanding its potential. Consequently, theatrical performance in the context of psychological intervention must address this constraint and, consequently, introduce resources to deal with it.

### A Metaphor of Theatrical Stage in Social Life

“All the world’s a stage,/And all the men and women merely players;/They have their exits and their entrances;/And one man in his time plays many parts” (Shakespeare: *As You Like It*, Act II Scene 7; *c*. 1603/2005, p. 52). The analogies between theater and everyday life, present in dialogues such as this well-known one from Shakespeare, illustrate the analysis of behavior in society as a manifestation of the theatricality – which is necessary and inevitable – existing in interpersonal relationships (analysis pioneered by Evreinoff, 2013). In this conception of theater as a social metaphor, two branches can be distinguished: one based on symbolic interactionism and the other having a Marxist or critical orientation.

The scope of *symbolic interactionism* ranges from the ideas of Mead (1972) about the roles existing in the configuration of the self to the Dramaturgical Perspective (Goffman, 1959) and its subsequent derivations. Symbolic interactionism has shown that we are all, simultaneously, actors and spectators in social life, to such as point that, for Goffman (Goffman, 1959, pp. 252–253), our self-image is in effect received from others (instead of the more elusive, real self). Specifically, self-image is understood as “some kind of image, usually creditable, which the individual on stage and in character effectively attempts to induce others to hold in regard to him. And the characteristic issue, the crucial concern, is whether it will be credited or discredited” (Goffman, 1959, p. 252). In other words, verisimilitude prevails over authenticity in the presentation of the self, which alerts us to the question of whether social situations – in general – tend to help show us as we are or, on the contrary, favor the genesis of strategies serving to present an alternative image of ourselves to others (see Walsh-Bowers, 2006, for a critique of this idea). As far as the Dramaturgical Perspective is concerned, these peculiarities of social interaction do not prevent individuals from maintaining the belief in a true or core self (Sullivan et al., 2014). As stated by Scheibe (2000, p. 227), “The dramaturgical perspective provides us with the keys for understanding why the problems of replication and the larger question of authenticity are so psychologically persistent.” Furthermore, this perspective offers a defense of the depth of everyday life – frequently disregarded – together with its capacity for transformation (Scheibe, 2017).

With its Theater of the Oppressed (Boal, 2009) the *Marxist or critical orientation* offers a practice aimed at emancipating or liberating individuals from dominant social structures, together with the obligatory development of an awareness of the dynamics of oppression. As Gergen (2012) has reminded us, “resistance to oppression must be embodied.” The Theater of the Oppressed has its origins in the theatrical pedagogy of Boal (2012) and one of the most popular branches is forum theater (Pestana and Codina, 2015a). In this, a member of the public is invited to re-enact the oppressed role from a previously observed scene. The interventions derived from the Theater of the Oppressed have even reached the business world (Meisiek, 2004; Meisiek and Barry, 2007), which confirms the popularity of this practice and its propagation in different fields.

Another proposal is F. Newman’s developmental theater, related to Marx’s thinking and also influenced by Vygotsky and Gergen. It considers that “the acting activity… is not an inner journey into a closed entity (either the character’s or the actor’s psyche); it is, instead, a social (interactive) journey into transformation” (Friedman, 1999, p. 177). Thus, the activity as a source of enlightenment or awareness takes precedence over the artistic purpose. Specifically, this practice draws attention to the efforts that people make to defend the strategies of the social institutions anchored in the self (Friedman, 1999). As a result of this discovery, individuals come to experience their own transformation.

In general, the traditions that have compared social life with theatrical performance have brought to light the diversity of resources that individuals – with a greater or lesser degree of self-consciousness – use to manage their personal relationships in the best way possible. However, two ideas overlie the metaphor of the theatrical stage in social life (Pestana, 2007): on the one hand, the inauthenticity or simulation that makes it difficult to access – or reveal – the essence of who we are; and on the other hand – and complementing the previous idea – the difficulties observed in certain social contexts when it comes to allowing individuals to experience who they are. In the words of Williams (2013, p. 95): “Thinking about authenticity in terms of dramaturgy draws attention away from its introspective aspects and refocuses instead on how authentic selves are expressed and negotiated in situations.”

### Theatrical Performance From an Alternative Perspective: A Leisure Experience

The presence of limitations both in theater as a psychological intervention technique (individual problems with the expression of the self) and in the metaphor of the theatrical stage in social life (inauthenticity linked to the details of the context) prompts the introduction of a perspective that can both complement the ones described above and add a method that serves to analyze the relationships between theatrical performance and the self: the leisure experience.

The leisure experience can be situated at a point between the clinical intervention and the social metaphor. This is explained by the fact that, on the one hand, it combines subjective elements related to the therapeutic and, on the other hand, the social dimension is fundamental to determining whether an activity can be considered leisure or not.

Research into the construct of leisure experience has led to a deeper understanding of the possible implications for human beings of leisure activities – as in the case of theatrical performance. For Kleiber et al. (2011, p. 100) the leisure experience corresponds to “the emotion that is experienced when leisure is recognized as being at hand, as it is apprehended,” understanding leisure as “a distinguishable context of relative freedom wherein preferred immediate experience has priority over instrumental outcomes… [considering freedom] not simply to be equated with choice or the lack of obligation but rather with the absence of worry and with a sense of opportunity and possibility” (idem). This experience is observed in particular when the activity is linked to the field of creation (Amigo and Cuenca, 2012).

With the incorporation of the experience into leisure research, we added to the analysis of what we do, the why and the what – specifically addressing the importance of perceptions of freedom (Iso-Ahola, 1980; Ellis and Witt, 1984) and satisfaction (Kleiber et al., 2011). In this way, the study of one of the main influences of leisure in the life of individuals – the development of self and identity – has been deepened (Kelly, 1983; Coleman and Iso-Ahola, 1993; Shaw et al., 1995; Kleiber, 1999; Kivel, 2000; Codina et al., 2017; Cuenca and Madariaga, 2017; Dattilo et al., 2018; Layland et al., 2018).

Nonetheless, that fact that an activity is experienced as leisure is not the only indicator determining whether it actually is leisure. Therefore, it is also important to consider the context in which it takes place. In the case at hand, it is obvious that theatrical performance is experienced differently depending on who takes part, whether they are professionals or not. However, in the first stage of training of performers – which is linked to self-knowledge, it can be observed that the relationship between leisure and self experience is related to the importance of freedom in the performer’s process of self-knowledge (Stanislavski, 1922/1967; Stanislavski, 1936). Whether freedom comes from the awareness of the dynamics of oppression (Chilcoat, 1998; Boal, 2009) or the individual’s ability to overcome his/her adherence to a single point of view about him/herself (Cruise and Sewell, 2000; Rowan, 2010), the perception of whether we are free – and if we are satisfied with what we do – can provide clues about what activities are more central to our self when we practice theatrical exercises.

To sum up – and as we understand it – the introduction of leisure experience as a factor in the analysis of theatrical performance makes it possible to overcome – at least potentially – the deficiencies observed in clinical intervention and the social metaphor (Pestana and Codina, 2017): specifically, by considering the self in a context that by definition offers greater freedom and satisfaction. With the empirical verification of this premise, the observation of two types of differences can be hypothesized: on the one hand, the differences between the various exercises that are part of a theatrical performance; and on the other, between these exercises and their association with the participants’ self-perception.

In other words, and in accordance with what has been exposed so far, this analysis brings with it a methodology that prioritizes the participants’ perception of themselves as regards who they are and what they do, and how these two aspects relate to each other when experiencing theatrical performance as a leisure experience.

## Materials and Methods

**FIGURE 2:**
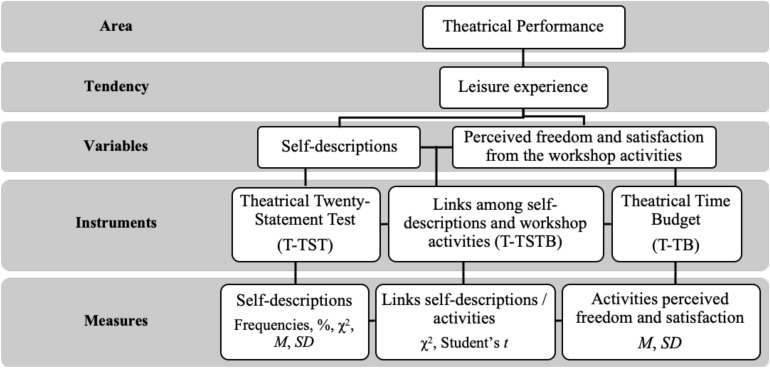
Main characteristics of the empirical study. Source: prepared by the authors.

In line with the classification made by Ato et al. (2013, p. 1,053), the empirical part of this research was developed through an observational study that meets all the requirements of a nomothetic and punctual kind. This implies that the data obtained was analyzed by means of a descriptive strategy, i.e., “the definition, classification and/or categorization of events to describe mental processes and overt behaviors” (authors’ translation). Figure 2 summarizes the main characteristics of this study.

### Participants

A group of 16 university students (15 women, one man) taking a degree in public relationships and aged between 18 and 21 years old (*M* = 19.06 years; *SD* = 1.06) cooperated in the study. They attended a theater exercises workshop that lasted three days (20 h). This was part of an optional credit (non-compulsory subject) on the aforementioned university degree syllabus. The participants gained a pass in this workshop through voluntary participation in the different proposed activities, so as not to establish differences according to performance in the different theater exercises and minimize the compulsory component of the workshop – thereby maximizing its potential as a leisure experience.

### Instruments

Data was collected using three instruments (all of them applied in the last part of the third day of the workshop).

The first was a version of the instrument known as the Twenty-Statement Test (TST: Kuhn and McPartland, 1967), which in its original form consisted of responding 20 times to the question “Who am I?” In the exploration of self and identity, the test offers the possibility of free expression without relinquishing the systematization of structured instruments (Codina, 1998). In the words of Rees and Nicholson (2004), it is “a qualitative research tool which can also yield codable and quantifiable assessments.” The validity of this test has been demonstrated by its recent use in research carried out in different contexts (Azghari et al., 2015; Escobar et al., 2015; Codina et al., 2017; Suslova, 2018), including joint analysis of theater and self-knowledge (Pestana and Codina, 2017). In the case of this research, respondents were asked for “twenty statements about yourself in this workshop” (i.e., theatrical self-descriptions), which is why the version of TST used here answers to the name of Theatrical TST (from hereon, T-TST).

Subsequent to the T-TST, a questionnaire with the structure and characteristics of the technique known as Time Budget was used. The TB was originally an instrument designed to record activities carried out at a given time (Andorka, 1987; Codina, 1999, 2004; Steinbach, 2006). Its introduction in leisure studies counted on the essential contribution of Neulinger (1986), who incorporated the evaluation of activities attending to psychological variables such as perceptions of freedom, satisfaction and intrinsic motivation, basic to the understanding of the leisure experience (Codina et al., 2016; Kleiber et al., 2017; Webb and Karlis, 2017). The TB used here – Theatrical Time Budget or T-TB – recorded the activities carried out during the three days of the workshop, specifying two valuations of them: participants’ perceptions of freedom and satisfaction (ranging from 0 to 100, from “not at all by choice/not at all satisfied” to “totally by choice/totally satisfied”) in each workshop activity.

Lastly, the third instrument – with the same T-TST layout – served for the participants to specify which theatrical activities in the workshop were most closely linked to their theatrical self-descriptions: “For each of the statements you wrote in the first questionnaire, indicate the activity in this workshop that you consider most closely linked to your answer. This consists of listing activities linked to the different statements about yourself” (for previous developments of this instrument, see Pestana, 2007; Pestana and Codina, 2015b, Codina et al., 2017). Thus, this last instrument highlighted the associations between self-descriptions and activities carried out, according to each participant’s point of view.

### Procedure

Before collecting data, we contacted the academic office of the university whose students would take part in the sample. After obtaining the corresponding authorization to use the applied instruments as a part of the research, the students were allowed to continue participating only if they agreed to sign the informed consent. The ethical requirements of the Ethics Committee of the University of Barcelona (University of Barcelona’s Bioethics Commission, CBUB – Institutional Review Board IRB00003099) were applied to the current study, which meant that additional approval for the research was not required because the data obtained did not involve animal or clinical experimentation. Additionally, this study complies with the recommendations of the General Council of Spanish Psychological Associations (Consejo General de Colegios de Psicólogos), the Spanish Organic Law on Data Protection (15/1999: Jefatura del Estado, 1999), and the Declaration of Helsinki (World Medical Association, 2013).

The categorization of self-descriptions followed the criteria established by Escobar et al. (2015). These authors drew on the analysis carried out by Kuhn and McPartland (1967) on the subjective meaning of the definitions that people provide about themselves (also called sub-consensual statements) to define four attitudinal categories (each with examples from the participants in this study): (1) *self-evaluations*, through which individuals express their way of being in the light of five possible dimensions –intellectual aptitudes (competencies that are not directly observable: “I’ve got to know myself better”), practical aptitudes (observable behavioral competencies: “I’ve learnt to control myself a little better”), character and morals (self-reflections: “I’m a creative person”), social life (relational characteristics: “I’ve experienced moments of closeness with strangers”), and emotional outlook (state of mind: “I’ve enjoyed myself”); (2) *self-esteem*, where people express their degree of satisfaction with themselves (“I’m less negatively self-critical”); (3) *preferences*, description of personal tastes (“I like facing challenges”); and (4) *ambitions*, statements regarding their own future (“I feel less afraid of the future”).

The theatrical activities in the T-TB were organized taking into account the following exercise categories (derived from sources related to theatrical training): relaxation, improvisation, objectives and obstacles and group discussion. *Relaxation* favors self-expression (Lelong, 1985; Guirchoun, 1995), and may even facilitate the emergence of unsuspected aspects and components of the self in those carrying out this activity (Strasberg and Hethmon, 1968). *Improvisation*, of proven utility in psychological interventions (Wiener, 1994; Lösel, 2019), is spontaneous behavior based on certain conditions, highlighting in the participants the degree of agreement (logic, coherence) between behavior and situation – with themselves and in relation to their peers, while stimulating various forms of physical and vocal expression (Brook, 1968, 1993; Johnstone, 1979; Strasberg, 1987). Furthermore, the value of improvisation as a way of conducting oneself in emergent, unpredictable and complex situations has been pointed out (Crossan et al., 1996; Sawyer, 1999). The notions of *obstacles* (which prevent a task from being carrying out) and *objectives* (the purpose that guides the actions being carried out) fuel the creative thinking needed to come up with novel solutions in distinctive situations (Knébel, 1996; Gutiérrez Bracho, 2017). In the words of Gené (1996, p. 44), “The actor does not usually need to know why his character does things. but it is certainly essential that the actor knows what he does something for” (authors’ translation). Lastly, *group discussion* encourages the participants to adopt an objective view of themselves in order to raise self-consciousness (Strasberg and Hethmon, 1968).

This description of activities is not intended to be comprehensive and the exercises are not mutually exclusive either. In general, having made a proposal for an exercise, its development and emphasis can be vary greatly depending on who carries its out. For example, in an improvisation it may be possible to experiment with different objectives or obstacles. Consequently, this classification of theatrical exercises should be understood as merely indicative, taking into account the main emphasis of the activity when presented to the participants. In any case, the selection of exercises takes into account the importance of theatrical activities “in analysing how the human being organises his own intersubjective relationships” (Sofia, 2013, p. 179).

The workshop schedule was organized as follows. The 20 h of the workshop were divided into two blocks of 7 h (during the first two days) and a block of 6 h on the third – and last – day. The start time was 10.30 a.m., with lunch from 1.30 p.m. to 3.30 p.m. After this break, the workshop continued until 6.30 p.m. (except on the third day, when it ended an hour earlier). Pauses of 5–7 min were included in each block of activities (morning and afternoon), depending on the dynamics of the activities carried out at the time and by agreement with the group.

The workshop began with a presentation and an exploration of the participants’ expectations, which led on to the activities. Regarding their distribution, each block began with relaxation followed by exercises involving objectives, obstacles and improvisations. At the end of each block, group discussion was used to find out about the participants’ experiences when carrying out the various activities in the block. As mentioned above, the information was collected at the end of the second block on the third day of the workshop, after which a final group discussion was held to take stock of the whole experience.

### Data Analysis

#### Self-Descriptions

In the case of self-descriptions registered by the T-TST, two data were obtained. The first concerned the prevalence of the categories used to classify the participants’ responses, i.e., the number *n* of participants who, out of the total of *N* = 16, refer to the type of self-description which each category refers to. To facilitate the understanding of the information, these frequencies are also presented in terms of percentages.

In order to assess whether the observed *n* of the categories was due to chance (or not), the Chi square coefficient (χ^2^) was calculated. In this coefficient, what was most important was the value ascribed to the probability *p* (ideally, *p* < 0.050), from which it is possible to reject the null hypothesis (Patten, 2005). In the case at hand (*N* = 16), if chance predominates, half the participants (*n* = 8) would be expected to present a type of categories and the other half would not. If this symmetry does not emerge, an absence of chance can be thought of as the cause of the phenomenon – in the case at hand, in the intervention carried out. When analysing the data obtained, it was taken into account that if 12 participants presented a category (and four did not), the value of χ^2^ would be the same as if four participants presented a category (and 12 did not). In this respect, the logic underlying this research was given priority, i.e., the manifestation of self-descriptions in the workshop – with the interpretation of what does not emerge in the self going beyond the scope of this study (interpretation more typical of a theoretical framework related to psychoanalysis). As a factor that adds precision to the description of this data, Chi square coefficient values are accompanied by the effect size provided by the value of Cramer’s *V*.

The second datum obtained about self-descriptions derives from how many times each participant mentions a specific T-TST category: specifically, the values of the corresponding means (*M*) and standard deviations (*SD*). In this way, it was possible to observe not only how many participants presented the categories of the self-descriptions, but also the mean of their responses in each category.

#### Theatrical Exercises

After calculating the Cronbach’s alpha value of the T-TB – we show the means (*M*) and standard deviations (*SD*) corresponding to the variables of the leisure experience: perceptions of freedom and satisfaction. As was to be expected, those exercises with the highest scores come closer to the leisure experience related to the development of the self.

#### Associations Between Self-Descriptions and Theatrical Exercises

These associations were analyzed in two ways. On the one hand, by looking at which exercises were significantly related to the categories of self-descriptions – and by using the Chi square coefficient. And on the other hand, by observing – with Student’s *t* (and its corresponding effect size shown by Cohen’s *d*) – whether the evaluations of the exercises are different according to whether or not there are categories of self-descriptions. In other words (and by way of an example), which exercise in the T-TST category of “practical” self-descriptions (related to acquired behavioral competencies) is perceived significantly as experienced more freely/satisfactorily? Answering this question implies assuming that if this description is to be fomented, the exercise closest to leisure experience should be given priority – also central in the development of the self.

## Results

In the case of the theatrical self-descriptions (Table 1), the presence of contents related to the acquisition of practical skills (χ^2^ = 12.25, *p* = 0.000, *V* = 0.88) and intellectual competences (χ^2^ = 9.00, *p* = 0.003, *V* = 0.75) was significant, as well as emotional outlook (χ^2^ = 6.25, *p* = 0.012, *V* = 0.63) and the expression of self-esteem (χ^2^ = 4.00, *p* = 0.046, *V* = 0.50). When observing means by category, the highest figures corresponded to the social (*M* = 5.88) and emotional categories (*M* = 3.56). When observing the mean of self-descriptions by category.

**TABLE 1:** Prevalence, means (*M*) and standard deviations (*SD*) of theatrical self-descriptions (*N* = 16).

	*n*	%	χ ^2^	*p*	*V*	*M*	*SD*
Intellectual	14	87.5	9.00	0.003	0.75*	2.50	1.75
Practical	15	93.8	12.25	0.000	0.88*	2.25	1.69
Character/moral	9	56.3	0.25	0.617	0.13	2.13	2.55
Social	16	100.0	6.50	0.090	0.64*	5.88	2.30
Emotional	13	81.3	6.25	0.012	0.63*	3.56	2.98
Self-esteem	4	25.0	4.00	0.046	0.50*	0.64	0.92
Preferences	9	56.3	0.25	0.617	0.13	1.00	0.96
Ambitions	7	43.8	0.25	0.617	0.13	0.63	0.80

*n refers to the number of participants whose self-descriptions had the category. The asterisk indicates values of Cramer’s V corresponding to a large effect size in the significant values of Pearson’s Chi Square. M and SD are related to how many times each participant mentions a specific category.*

The T-BT with the list of theatrical exercises carried out during the workshop obtained a Cronbach’s alpha of 0.877, which demonstrates the internal consistency of this instrument. Regarding the evaluations of the activities, of the five types of theatrical exercises developed throughout the workshop (Table 2), the group discussion was the activity with the highest perception of freedom (*M* = 78.67, *SD* = 23.63), followed by obstacle exercises (*M* = 66.43, *SD* = 24.98). On the other hand, the activities whose purpose was to achieve an objective were the theatrical exercises with a lower perception of freedom (*M* = 54.53, *SD* = 29.11). As regards the perception of satisfaction, the highest value was observed in the relaxations (*M* = 81.33, *SD* = 23.48); on the contrary, the exercises focusing on objectives were those perceived as less satisfactory (*M* = 53.63, *SD* = 23.81).

**TABLE 2:** Means and standard deviations for perceptions of freedom and satisfaction of theatrical activities practiced (*N* = 16).

	Freedom		Satisfaction	
Theatrical activities	*M*	*SD*	*M*	*SD*
Relaxation	62.32	42.53	81.33	23.48
Improvisation	62.88	26.69	64.88	21.31
Objectives	54.53	29.11	53.63	23.81
Obstacles	66.43	24.98	65.76	15.32
Group discussion	78.67	23.63	68.33	19.79

By associating the presence of theatrical self-descriptions with the activities of the workshop – data not tabulated – it was observed that among the 16 participants who provided theatrical self-descriptions with relational (social) characteristics, eight associated these self-descriptions with improvisations, four with group discussions, three with obstacle exercises and one respondent with objective-based activities (χ^2^ = 6.50, *p* = 0.090, *V* = 0.64).

## Discussion

In this paper we present the bases of the conception of theatrical performance as leisure experience, an approach that can complement the metaphor of the theatrical stage in social life and the use of theater as a technique in psychological intervention. Given that in the leisure experience freedom and satisfaction are central to a more comprehensive understanding and development of the self, interventions that use this alternative approach may provide a way of overcoming the limitations represented – at least potentially – by difficulties in expressing or empowering the self, or by the lack of authenticity linked to the circumstances of the context.

The conception of theatrical performance as leisure experience, instrumentalized by means of a workshop of theatrical exercises, offers promising results. It was observed that the exercises used in a theater workshop offer differentiated subjective experiences with respect to freedom and satisfaction. In other words, in an intervention carried out with theatrical exercises, each activity receives a specific assessment that must be taken into account. Regarding the results of the study carried out, the relevance of exercises such as group discussion (as noted by Strasberg and Hethmon, 1968), obstacles (Knébel, 1996) and relaxation (insufficiently worked on as a personal experience according to Kleiber, 2000) should be highlighted. These stand in contrast to the scores obtained for the exercises focusing on objectives, which, although they were rated lower (within the set of workshop activities), offer the opportunity to experiment with directionality in this line of work.

Freedom and satisfaction, as the basis of the leisure experience, also affect the development of the self and identity (as recently noted, among others, by Codina et al., 2017; Dattilo et al., 2018; Layland et al., 2018). Regarding the self-descriptions linked to the theatrical performance, the centrality of the acquisition of competences – practical and intellectual – can be observed in the answers obtained. Thus, the intervention carried out specifies what characteristics of the self are manifested in a context of theatrical activities as a leisure experience. Furthermore, the participants pointed out the associations between self-descriptions and theatrical exercises: If we know what exercises mobilize certain aspects of the self, then psychological and social intervention with a theatrical base can be more enriching for the participants and more useful to researchers.

It is worth highlighting the relevance of the instruments used to obtain this data. Thanks to the combined application of TB (in line with Neulinger, 1986) and TST (originally proposed by Kuhn and McPartland, 1967) to theatrical performance, it is possible to specify relationships that deserve consideration in future workshops and interventions.

The arguments presented here in favor of theatrical performance as a leisure experience supporting the development of the self are susceptible to dialogue with other theoretical frameworks. By way of an example, the empirical approach proposed in this paper could be useful for identifying – in the self-descriptions themselves – psychic complexes, cognitive abilities, psychodramatic roles, self-image traits and embodied oppressions. In other words, the empirical research carried out in this study can also offer an instrumentalization suited to the analysis of theatrical performance as a clinical intervention and as societal metaphor.

This analysis of our findings does not ignore the limitations of the study carried out. The very core of the concepts worked here – being sensitive to both individual and social aspects – requires further studies to consolidate the findings of this research. In this respect – and by way of an example, if we were to use theatrical exercises different from those used in this study (as proposed by Olenina et al., 2019), or different taxonomies of self-descriptions, it might be possible to deepen the influence of theatrical performance – conceived as leisure experience – on the self. Likewise, the use of interventions of variable length, accompanied by their corresponding evaluation and monitoring in different samples, could serve to specify the type and characteristics of intervention programmes – as well as their results.

Whatever the case, any aspects that converge in the arena of behavior will find references in freedom, satisfaction and self that enhance the importance of the theatrical performance as leisure experience, when answering fundamental questions about the meaning of human existence.

## Data Availability Statement

The datasets generated for this study are available on request to the corresponding author.

## Ethics Statement

The ethical requirements of the Ethics Committee of the University of Barcelona (University of Barcelona’s Bioethics Commission, CBUB – Institutional Review Board IRB00003099) were applied to the current study, which meant that additional approval for the research was not required because the data obtained did not involve animal or clinical experimentation. Additionally, this study complies with the recommendations of the General Council of Spanish Psychological Associations (Consejo General de la Psicología de España), the Spanish Organic Law on Data Protection (15/1999: Jefatura del Estado, 1999), and the Declaration of Helsinki (World Medical Association, 2013). The patients/participants provided their written informed consent to participate in this study.

## Author Contributions

JP conceived and designed the research for this manuscript. He was also responsible for drafting the whole work and revising it critically for important intellectual content. RV was responsible for the analysis of data gathered during the research, revising it critically with the purpose of improving its explanatory potential. NC was responsible for the analysis and interpretation of data gathered during the research, revising it critically for important intellectual – theoretical and methodological – content. All authors contributed to the article and approved the submitted version.

## Conflict of Interest

The authors declare that the research was conducted in the absence of any commercial or financial relationships that could be construed as a potential conflict of interest.
